# The Influence of Biological Age and Sex on Gross Motor Skill Development in Young Athletes: A Pilot Study

**DOI:** 10.3390/sports14040153

**Published:** 2026-04-15

**Authors:** Matthew S. Chapelski, Tyler Tait, Stacey Woods, Sarah Benson, Marta C. Erlandson, M. Louise Humbert, Adam D. G. Baxter-Jones

**Affiliations:** College of Kinesiology, University of Saskatchewan, Saskatoon, SK S7N 5B2, Canada; m.chapelski@usask.ca (M.S.C.); tyler.tait@usask.ca (T.T.); srw051@mail.usask.ca (S.W.); sarah.benson952@usask.ca (S.B.); marta.erlandson@usask.ca (M.C.E.); louise.humbert@usask.ca (M.L.H.)

**Keywords:** athletes, youth, gross motor skills, maturation, biological age, sex

## Abstract

**Background:** Gross movement skills (GMS) development is important for long-term physical activity participation. Despite this, the influence maturation has on GMS is understudied. The purpose of this pilot study was to evaluate the effect of maturation and sex on GMS in adolescents and identify numbers for a definitive study. **Methods:** We recruited seventy-one athletes (21 male, 50 female) from 8 to 17 years of age. Height, sitting height, and body mass were measured, and biological age (indexed as years from peak height velocity [PHV]) was predicted. Athletes were classified into three maturational categories: pre-PHV, peri-PHV, and post-PHV. The Test of Gross Motor Development-2 was used to assess GMS. Differences in overall GMS, locomotor skill, and object control skills were evaluated using ANCOVA controlling for height, weight, sex, physical activity, and sport specialization. **Results:** We found that GMS scores were greater for athletes post-PHV (83.62 ± 6.09) when compared to athletes peri-PHV (74.25 ± 12.92; *p* = 0.01). There were no differences between the pre-PHV and post-PHV groups (*p* = 0.13). Between sexes, males had greater GMS scores than females within each maturational category (*p* < 0.05). **Conclusion:** Our pilot study is inconclusive but suggests that factors such as sex, exposure to different GMS, and time spent practicing GMS may influence GMS performance to a greater extent than maturation. However, these findings are underpowered; a sample of 154 would be required for a definitive study.

## 1. Introduction

Gross-motor skills (GMS) involve large movements of the trunk, arms, and legs that result in achieving a motor task such as jumping, throwing, or running [1]. It is well documented that GMS proficiency increases with chronological age [2,3,4] and has a positive correlation with physical activity (PA) participation from 2 to 15 years of age [5]. Furthermore, individuals who have fully developed GMS can participate in PA with ease, whereas restricted GMS development tends to prevent individuals from participating in PA and precludes them from further GMS development [6,7,8,9]. This has been described as the proficiency barrier; thus, GMS lays the foundation for the development of sport-specific skills that are required for athletic success and longevity [8,10,11]. Theoretical models of GMS development tend to indicate that GMS are fully developed around 7 to 10 years of age [10,12]. After this point, adolescents tend to be engaged with sport-specific motor skills associated with their athletic pursuits [10,12].

A systematic review by Sliedrecht et al. found that athletes competing for high-performance teams (those selected or playing in higher divisions) had better GMS proficiency than other athletes competing at a recreational level [13]. Furthermore, GMS proficiency in child and adolescent athletes has been associated with exposure and practice time [14,15]. Specifically, those receiving more practice time and exposure to different GMS have better GMS proficiency. Some athletes specialize in a single sport before adolescence (before 11 years of age) [16]. Single-sport practice has been found to negatively impact overall GMS development, as these athletes are exposed to a limited array of GMS [17,18]. This is crucial, as GMS has a positive relationship to PA [5], and adolescence is when the formation of adult PA habits occurs [19,20].

Adolescence is characterized by changes in body size, fat, and muscle mass [19,21,22,23,24]. Furthermore, from 6 to 16 years of age, strength (positive effect) and fat mass (negative effect) are known to influence GMS [25,26,27]. Adolescence is also characterized by increasing maturity that affects size. However, most studies lack a measurement of maturity, which limits the ability to account for individual chronological age developmental differences that may influence GMS, as individuals can differ in biological age at similar chronological ages. In young children (7 to 9 years of age; pre-pubertal), Salami et al. [4] found that chronological age rather than biological age was positively associated with GMS in children 7 to 9 years of age. Similarly, in a sample of 429 children 7 to 10 years of age, Freitas and Colleagues found that biological (skeletal) age was not associated with gross motor development [28]. In contrast, others have found that GMS increases with biological age in older children (peri- and post-pubertal) from 10 to 17 years of age [29,30]. Differences between studies may be due to the disparities in the age range assessed, the assessment of biological age, or the tool used to evaluate GMS development.

In athletic populations, maturation interacts with training loads [31] and sport-specific movement patterns [32]. Thus, as athletes mature, changes in muscle mass, limb length, and body mass [19,21,22,23,24] can alter the effects of structured training. This means that maturing athletes may display accelerated gains in strength, power, and movement efficiency as maturation enhances their responsiveness to the technical and physical demands of their sport. This highlights the need to understand the influence of maturation on GMS development in athletes, as the studies currently available examine only habitually active children.

One of the other main contributors to GMS proficiency is sex. Specifically, before puberty, females tend to have better GMS proficiency, especially in balance and coordination skills (e.g., hopping) [33]. However, during puberty, males tend to have greater overall GMS proficiency [33,34]. It has been suggested that these sex-related differences in GMS are likely driven by differences in muscle development. It is unclear if these sex-related differences in GMS development are present in young athletes.

The purpose of this pilot study is to evaluate whether GMS proficiency differs between young athletes in different maturational states (pre-, peri-, and post-pubertal) and between sexes. We hypothesized that more mature individuals would have a higher GMS score due to their greater size and strength. Furthermore, we hypothesized that males will have higher GMS than females. Given the exploratory nature of the study, the hypotheses are simplified representations of the complex developmental interactions that can influence athletes’ GMS.

## 2. Materials and Methods

### 2.1. Study Design

This study used an observational cross-sectional design. We defined sex as a set of biological, physical, and psychological attributes. In 2017, seventy-one participants, 8 to 17 years of age (50 female), were recruited from the University of Saskatchewan summer activity camps and sports programs from around the City of Saskatoon. All participants were engaged in at least one sport (52% were involved in 2 or more sports). A table displaying the recruitment sample size by sports program can be found at Supplemental Table S1. To participate in the study, children needed to be healthy and have no history of mental and/or physical conditions that would impair their GMS performance. Ethics approval was obtained through the University of Saskatchewan Behavioural Research Ethics Board (BEH: 16-160; 16 May 2016). Before participating in the study and signing the consent and assent forms, a thorough explanation of the study procedures was provided to all participants and their parents/guardians. All procedures were performed at the University of Saskatchewan during camp break or rest periods, or after camp was completed for the day. Measurements were completed by one individual at a time to reduce the influence of others on the study protocol (e.g., seeing how others complete the GMS Test).

### 2.2. Anthropometry

Anthropometric measurements included height, sitting height, and body mass. Height and sitting height were measured to the nearest 0.1 cm using a portable stadiometer (Seca, Hamburg, Germany), while body mass was measured to the nearest 0.1 kg using a Tanita scale (Model 1631, Tanita Corp., Tokyo, Japan). Height, sitting height, and body mass were measured twice and had to be within 0.5 cm or 0.1 kg, or a third measurement would be taken; the mean of the two closest measurements was used for analysis. Body mass and height were used to calculate body mass index (BMI). Absolute BMI was then compared against CDC reference data [35] to calculate an age-relative percentile for each participant.

### 2.3. Biological Maturity

Maturity or biological age was assessed using a sex-specific multiple regression equation, developed by Mirwald et al. [23], which predicted the participant’s years from attainment of peak height velocity (PHV). This equation uses chronological age, height, sitting height, weight, and leg length measurements to estimate the age of PHV (APHV). APHV was used to group participants in one of three maturity groups: pre-PHV, which was defined as <−1 year from the average age at PHV; peri-PHV, which was defined as >−1 year from average PHV and <1 year after average age of PHV; post-PHV, which was defined as >1 year after the average age of PHV [36]. It has been suggested to use these maturational categories since most maturity indicators, including the Mirwald equation, have a standard deviation of one year [37].

### 2.4. Gross Motor Skill Score

Participants’ GMS was evaluated using the Test of Gross Motor Development-2 (TGMD-2), which has been found to be a valid and reliable tool for assessing GMS in adolescents [38]. TGMD-2 is composed of two subtests (locomotor and object control) that measure motor proficiency of six locomotor and six object control skills, and a complete list of skills can be found in the TGMD-2 testing manual [39]. For each task, participants were told what motor skill they would be executing, provided a demonstration, and were allowed to practice if desired. All TGMD-2 assessments were conducted on a standardized indoor gymnasium surface in alignment with TGMD-2 guidelines. For each participant, all skills were video recorded for scoring later. Each participant completed each task twice. After all testing was completed, all trials were scored in accordance with the TGMD-2 protocol. Specifically, each trial was scored on a scale from 0 to 1, with 1 indicating correct movement behaviors and 0 indicating incorrect movement behaviors for a given skill. For each skill, the score from the two trials was combined to obtain a raw score. Each child was scored by one of four trained coders. Inter-rater reliability (IRR) was tested on the four coders (IIR = 0.71) and fell within the acceptable range for this tool [40]. The raw scores of individual skills were then added together to create an overall gross motor quotient ranging from 0 to 100. A higher GMS score indicates greater motor proficiency.

Since this pilot study was completed in 2017, the TGMD-2 was used as it was the most recent version of the tool. However, since then, the TGMD-3 has been developed. A comparison between these tools in young children suggested these tools should not be used interchangeably [41].

### 2.5. Physical Activity Measurement

PA levels were assessed using the validated Physical Activity Questionnaire for Older Children (participants from 8 to 14 years of age) or Adolescents (participants from 14 to 18 years of age) [42,43,44,45]. This 7-day recall self-report questionnaire provides information regarding general PA participation. The questionnaires use a 5-point Likert scale with a rating of one being low levels of PA and five being high levels of PA.

### 2.6. Sport Specialization Measurement

Sports specialization was determined using the Adolescent Physical Activity Recall Questionnaire (APARQ), which is valid and reliable [46]. The APARQ is a 1-year recall questionnaire that assesses both organized and non-organized activity during a typical week [46].

### 2.7. Statistical Analysis

All statistical analyses were performed using IBM SPSS version 29.0 (IBM Corp., Armonk, NY, USA). Normality was assessed using the Kolmogorov–Smirnov test. All variables were normally distributed except height, maturity offset, and APHV. Log and square-root transforms did not correct the frequency distribution; therefore, statistical differences were assessed using the raw data. To assess the normality of residuals, we examined a Shapiro–Wilk test. The results indicated that the residuals were not normally distributed for GMS in the pre-PHV group [Shapiro–Wilk = 0.876, *p* = 0.012]. The normality of residuals for all other groups was normally distributed (*p* > 0.05). Homogeneity of variance was tested using Levene’s Test. The test was not significant [F(2,71) = 0.172, *p* = 0.842], indicating that the variances across groups were equal. Finally, to test the homogeneity of regression slopes, we evaluated the interaction between the covariates and the biological age groups. The interaction term was significant for biological age group and sex [F = 6.576, *p* = 0.001] and biological age group and sport specialization [F = 3.010, *p* = 0.038]. All other interactions were non-significant.

An Analysis of Covariance (ANCOVA) was used to assess differences in height, body mass, BMI, PA, and sports specialization between maturity groups, while controlling for the effect of sex. Similarly, an ANCOVA was also used to assess differences in GMS score between maturity groups, while controlling for height, weight, sex, PA, and sports specialization. Statistical differences were indicated when *p* < 0.05. Data are presented as means and standard deviations.

Statistical analysis revealed we obtained a partial eta (η^2^) of 0.12 and a power of 0.83. We calculated that a sample size of 154 (around 52 athletes per maturational category) would be needed to validate the findings of this study [47].

## 3. Results

Table 1 displays the participants’ demographics. The post-PHV group was significantly (*p* < 0.05) older, taller, heavier, and more mature than the peri- and pre-PHV groups. Similarly, the peri-PHV group was significantly (*p* < 0.05) older, taller, heavier, and more mature than the pre-PHV group. BMI percentile was not different between maturational categories (*p* = 0.18). The APHV was significantly older in the post- compared with the peri- and pre-PHV groups, suggesting that the timing of maturation was later in the post-PHV group. For PA, there was a main effect of maturational category [F(2,71) = 6.43, *p* = 0.003, ƞ^2^ = 0.16]. Pairwise comparisons indicate that the PA score is significantly greater (*p* = 0.004) for the pre-PHV compared to the post-PHV group only. For sports specialization, there were no differences (*p* = 0.38) between maturational categories.

**Figure 1:**
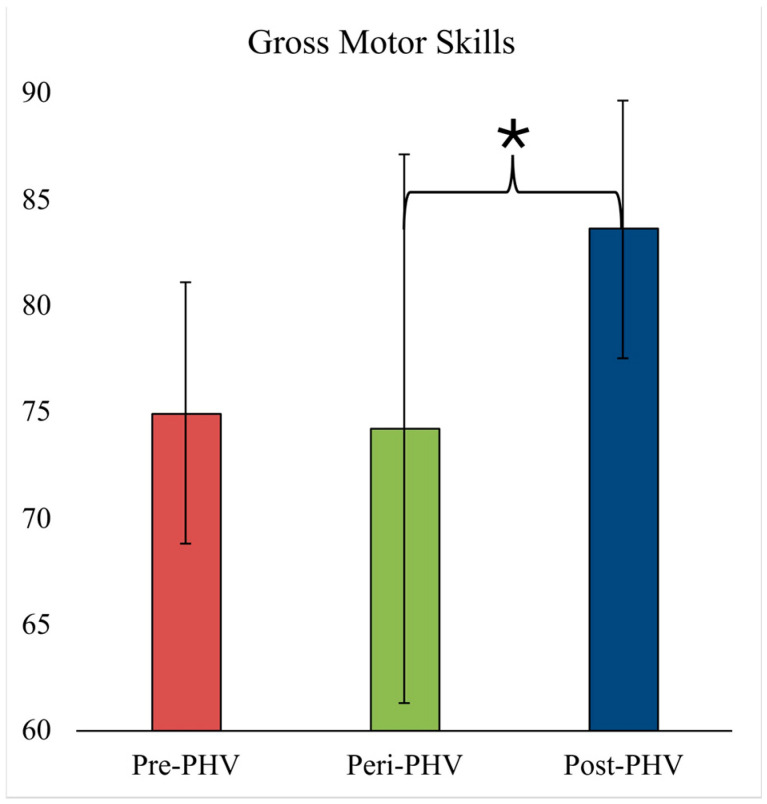
Gross motor score for each biological age group. * Significant difference between peri-PHV and post-PHV. Abbreviations: PHV—peak height velocity.

Figure 1 displays the overall GMS score for each maturational category. Overall, GMS scores were significantly different between maturational categories [F(2,71) = 4.62, *p* = 0.013, ƞ^2^ = 0.13, observed power = 0.745] even when controlling for differences in height, body mass, sex, PA, and sport specialization. Pairwise comparisons indicate that GMS scores were significantly lower in the peri-PHV group compared to post-PHV groups (*p* = 0.01), and not different between pre-PHV and peri-PHV groups (*p* = 1.00) or between pre-PHV and post-PHV groups (*p* = 0.13). Sex [F(2,63) = 8.063, *p* = 0.006, ƞ^2^ = 0.113, observed power = 0.798] was the only significant covariate factor. This proposes that the relationship between the overall GMS score and the maturational category may be different between sexes. Specifically, we found the GMS score was greater in males compared with females (Supplemental Table S2).

**Figure 2:**
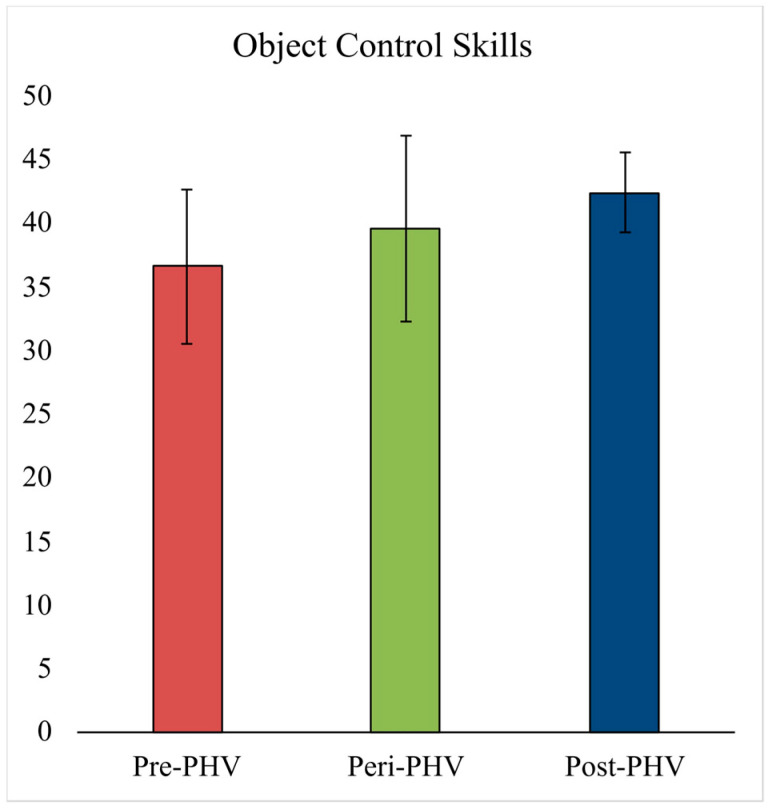
Object control score for each biological age group. Abbreviations: PHV—peak height velocity.

**Figure 3:**
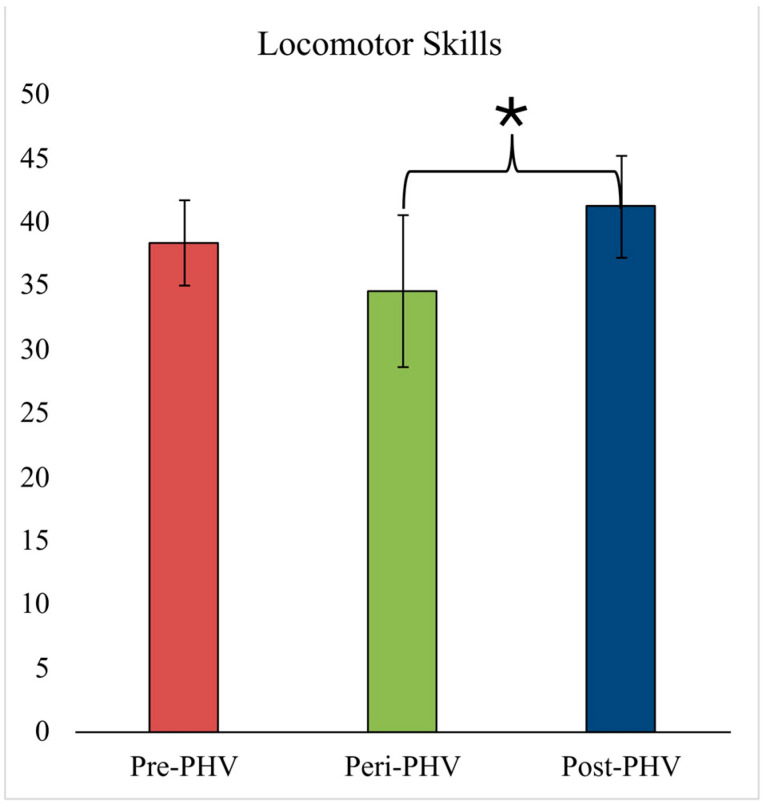
Locomotor score for each biological age group. * Significant difference between peri-PHV and post-PHV. Abbreviations: PHV—peak height velocity.

The object control and locomotor score for each maturational category are presented in Figure 2 and Figure 3, respectively. After controlling for differences in height, body mass, sex, PA, and sport specialization, only the locomotor score was significantly different between maturational categories [F(2,71) = 8.21, *p* = 0.001, ƞ^2^ = 0.21, observed power = 0.951]. Specifically, the locomotor scores were significantly lower in the peri-PHV group compared to post-PHV groups (*p* = 0.002), but not different between pre-PHV and peri-PHV groups (*p* = 1.00) or between pre-PHV and post-PHV groups (*p* = 0.51). The object control score was not different between groups (*p* = 0.44).

## 4. Discussion

The aim of this pilot study was to evaluate differences in GMS between youth athletes in pre-, peri-, and post-PHV biological age groups and to estimate numbers required for a definitive study. In accordance with our hypothesis, we observed that the GMS score was greater in athletes post-PHV compared to peri-PHV. We found no differences between the pre-PHV and post-PHV groups. Overall, we found that the GMS score was likely affected by sex, with males having higher scores than females across all maturational categories. Although our sample’s distribution of males and females is uneven (21 males, 50 females). When dividing the GMS score into subsets of locomotor and object control, we found that differences in overall GMS between sexes and maturational categories could be driven by differences in locomotor skills. Power calculations showed a definitive study required 154 athletes and indicated our study was underpowered. To make definitive sex differences, a sample of 154 male and 154 female athletes would be required.

It is not surprising that the post-PHV group was taller and heavier compared with their less mature counterparts. As mentioned previously, adolescence is characterized by rapid growth, which results in increases in body size, muscle mass, and strength [19,21,22,23,24], which may explain the higher GMS score in the post-pubertal group. Greater muscle strength can allow an individual to better manipulate their body and external objects with greater ease [2,48]. In contrast to our results, Drenowatz and Greier found that early maturing children (post-PHV) had lower motor competence than children who were late maturing (pre-PHV) [49]. They also found that early maturing children had greater weight [49], which is associated with lower motor competence [27,50]. However, their study organized maturation based on sex-stratified tertiles and recruited from middle schools [49], while we organized maturation into categories and recruited athletes. In our case, we organized our sample based on fixed categories, whereas tertiles would divide participants into equal-sized groups based on their distribution and not their maturational grouping (i.e., they could all be the same maturational group), which influences group composition and comparisons. Furthermore, with our sample being primarily athletes, the lack of differences is probably influenced by a ceiling effect where our athletes had already advanced to the sport-specific phase of motor development [10]. In other words, measuring differences in GMS could be challenging because athletes’ motor skill acquisition is already highly developed, which leaves little variation to detect differences due to maturation.

We found that GMS did not differ between pre- and peri-PHV groups as well as pre- and post-PHV groups. In combination with our interactions, it proposes that other factors besides maturity may affect our GMS results. Although a certain level of maturation may be required to learn and perform some motor skills, most GMS are learnt before puberty [10,51,52]. Therefore, it is possible that participants already had a high level of motor skill development, and to detect differences, mastery of the skills would be required.

In relation to motor development models like the Long-term development in sport and physical activity 3.0 [12] or the Mountain of Motor Development [10], our athletes would be exposed to more sport-specific motor skills. Thus, our athletes likely mastered the GMS measured in this study. Again, the lack of differences highlights a potential ceiling effect where differences between biological age groups are so small they are undetectable. Namely, there was only a 0.7-point and 9.4-point difference between pre- and peri-PHV groups, and pre- and post-PHV groups, respectively. Additionally, the GMS score was high among all maturational categories (Score > 75). Together, these findings and theoretical GMS development pathways support the idea that athletes have a greater focus on GMS mastery and sport-specific skills development during adolescence [20,53].

In the present study, we found no differences in the sport specialization score, which indicates our groups have similar levels of sport exposure and could explain why there were minimal differences between groups. Another influential factor that could explain the lack of difference in GMS between pre- and post-PHV groups could be physical activity exposure. The physical activity score was significantly higher in the pre-PHV group compared with the post-PHV group, suggesting that the pre-PHV group may have more time to practice GMS [27,48]. Furthermore, given our pre-PHV group is younger than both the peri- and post-PHV group, they could be exposed to more GMS rather than sport-specific skills based on theoretical models of motor development [10,12]. This highlights the need for future studies to expand the sample size and consider more factors that can influence GMS development.

When subdividing the TGMD-2 into locomotor and object control arms, we only found significant maturational category differences for locomotor motor skills. This could be due to the sports we recruited from, which consisted of sports that expose participants to more object control (e.g., soccer) development rather than locomotor (e.g., dance). Thus, the participants’ sports background may account for the difference between the post-PHV and peri-PHV. This may also account for the absence of differences between the post-PHV and pre-PHV groups. Moreover, this highlights potential sampling bias as some sports’ specialized skills still emphasize fundamental motor skills (e.g., kicking a soccer ball versus stick-handling a puck). Finally, females have greater locomotor competence during childhood [54,55,56], which could exist in adolescence; thus, our primarily female sample could explain these differences.

Of the many covariates included in the model, sex was our only significant covariate. This is not surprising as males have been found to have greater motor competence when compared to females [29,57,58]. Specifically, males have greater motor competence for running [59] and object control [57,59,60,61,62,63,64]. Furthermore, it was found that maturation increases the difference in GMS between males and females [58]. Finally, skeletal maturation explains 8.1% of the variance in males’ GMS and 2.8% of females’ GMS at 11 to 14 years of age [28]. These findings highlight the impact sex could have on our findings. Even though all three maturational categories are mostly females, our findings still highlight that GMS development is different between males and females.

### 4.1. Limitations

A central limitation of this study is the small sample size and low statistical power, which is compounded when we divided the athletes into maturational groups. The uneven distribution across maturity groups may also have amplified the influence of individual variability, making the peri-PHV estimates less stable than those of the pre- and post-PHV groups. The unequal sample size across maturational categories can reduce the statistical power of the analyses. Disproportionate group sizes may skew comparisons and affect the robustness of differences between subgroups. This approach has led to our study being underpowered for the given analysis.

Another limitation is the small sample size; we removed 13 participants from inclusion in the study since they lacked full questionnaire data. Although we had 71 participants, a power analysis showed we required 52 per group. When the 13 participants were added to the study, and we compared PHV groups using an ANCOVA while controlling for height, weight, and sex, we found the post-PHV group had greater GMS (*p* = 0.030) and object control (*p* = 0.008) compared to the pre-PHV group. Supplemental Table S3 contains this sample’s data and demographics from the analysis.

In addition, the use of the Mirwald equation introduces potential error in maturity classification. Although widely used, this predictive equation is known to misclassify individuals by ±1 year or more, particularly the further they are away from their PHV [23]. Such a classification error may have attenuated true maturity-related differences or contributed to overlap between groups. Together, these factors warrant caution when interpreting the magnitude of maturity effects observed in this pilot study. However, sorting our population by biological age categories rather than equal tertiles is more accurate since we are examining maturational groups, and categorical sorting allows us to align participants based on meaningful developmental stages rather than tertile cutoffs.

The majority of children in our sample were already physically active and regularly involved in organized sports or recreational activities. This relatively high baseline level of activity may have limited the variability within the sample and, as a result, could have masked or diminished the observable effects of the maturation.

The cross-sectional nature of the project prevents the observation of individual variability over time. This design captures data at a single time point, making it impossible to examine changes in GMS across the maturational process.

### 4.2. Practical Application

Although underpowered, the findings of this study provide insight for coaches and practitioners working within the GMS development. First, the finding that post-PHV athletes demonstrated higher GMS scores than peri-PHV athletes, but not higher than pre-PHV athletes, suggests that maturation alone is not a consistent driver of GMS proficiency. Practitioners should therefore avoid assuming that older or more biologically mature athletes will naturally demonstrate more advanced GMS. Instead, training environments should emphasize structured, progressive exposure to a wide range of locomotor and object control skills. Moreover, the study’s findings underscore the importance of intentional skill practice over passive maturation. In other words, GMS does not seem to be something that naturally increases during maturation. GMS development should incorporate repeated practice opportunities that emphasize motor skill refinement. Embedding these principles into physical education curricula, community sport programs, and early athlete development pathways can support more consistent skill acquisition regardless of maturation timing.

Second, we found sex differences favored males within each maturational category, which is consistent with previous sex-difference GMS research. Coaches and educators can address these gaps by ensuring that females receive equitable, varied, and developmentally appropriate opportunities to practice object control, as this tends to be an area where disparities often emerge between males and females.

Although our study is underpowered, it highlights that skill exposure, practice, and equitable instructional design may be more influential than biological maturation alone in shaping gross movement skill development across adolescence.

## 5. Conclusions

Although inconclusive, our pilot study suggests the GMS score may be higher for post-pubertal athletes compared to the athletes who were going through maturation. However, our findings are affected by an uneven distribution across maturity groups within our sample; thus, one should exercise caution when interpreting these results. In athletes, other factors such as sex and exposure to motor skills may contribute to variance in GMS proficiency rather than maturation. A future research project with a sample size of 154 is needed to verify this idea.

## Figures and Tables

**Table 1:** Participant descriptives.

	Maturity Group		
	Pre-PHV	Peri-PHV	Post-PHV
N (% females)	21 (60%)	8 (63%)	42 (78%)
Age (years)	9.34 ± 1.23	12.30 ± 1.22 *	15.36 ± 1.22 *^†^
Height (cm)	136.09 ± 6.14	152.58 ± 6.95 *	167.08 ± 4.57 *^†^
Body Mass (kg)	32.66 ± 6.18	45.60 ± 6.95 *	61.65 ± 8.35 *^†^
APHV (years)	12.15 ± 0.77	12.63 ± 0.81	13.39 ± 0.88 *
Maturity Offset	−2.81 ± 1.00	−0.32 ± 0.51 *	1.97 ± 0.55 *^†^
Physical Activity Score	3.18 ± 0.71 ^µ^	3.07 ± 0.41	2.64 ± 0.44
Sports Specialization Score	1.71 ± 0.56	1.63 ± 0.52	1.52 ± 0.51

* Significantly different from pre-PHV. ^†^ Significantly different from peri-PHV. ^µ^ Significantly different from post-PHV. Abbreviations: age of peak height velocity (APHV), peak height velocity (PHV).

## Data Availability

The data presented in this study are available on request from the corresponding author. The data are not publicly available due to ethical restrictions.

## Supplementary Materials

The following supporting information can be downloaded at: https://www.mdpi.com/article/10.3390/sports14040153/s1, Table S1: Recruitment Information; Table S2: Participant Descriptives and TGMD-2 Scores divided by Sex; Table S3: Participant Descriptives and TGMD-2 Scores for additional analysis.

## Abbreviations

The following abbreviations are used in this manuscript:

GMS	Gross Motor Skills
PA	Physical Activity
PHV	Peak Height Velocity
TGMD-2	Test of Gross Motor Development-2
