# Supplementary material for: The Influence of Biological Age and Sex on Gross Motor Skill Development in Young Athletes: A Pilot Study
Source: Sports (Basel). 2026 Apr 15;14(4):153. doi: 10.3390/sports14040153 (PMC13119707; doi:10.3390/sports14040153)
Supplement: Supplementary file 1 [file sports-14-00153-s001.zip › sports-4209451-supplementary.pdf]

Supplemental Tables

Supplemental Table S1. Recruitment Information

Program	Trial Run	Activity Camps	Varsity Sports Camps	Soccer Program	Swim Program	Synchronized Swim Program	Hockey Program
Population	4	800	120	40	100	90	14
Available Participants	4	200	120	36	80	60	14
Participants Recruited	4	11	6	36	7	8	12
Percentage Recruited	100%	6%	5%	100%	9%	13%	86%

Supplemental Table S2. Participant Descriptives and TGMD-2 Scores divided by Sex

	**Males** (n=21)		
	**Pre-PHV** (n=9)	**Peri-PHV** (n=3)	**Post-PHV** (n=9)
GMS	76.22±2.91*	85.33±4.93*	86.44±2.60*
Object Control	37.22±3.38	39.00±4.58	42.11±2.42
Locomotor	39.00±3.54	46.33±1.15	44.33±1.94
	**Females** (n=50)		
	**Pre-PHV** (n=12)	**Peri-PHV** (n=5)	**Post-PHV** (n=33)
GMS	74.00±7.76	67.6±11.5	82.85±6.55
Object Control	39.17±3.19	32.00±5.34	40.97±4.33
Locomotor	34.83±7.04	35.6±6.19	41.88±3.20

*Significant differences between sexes for that biological age group

Abbreviations: age of peak height velocity (APHV), peak height velocity (PHV)

Supplemental Table S3. Participant Descriptives and TGMD-2 Scores for additional analysis

	**Maturity Group**		
	**Pre-PHV**	**Peri-PHV**	**Post-PHV**
**N** (% females)	21 (57%)	12 (75%)	51 (78%)
**Age** (years)	9.34±1.22	12.42±1.08*	15.39±1.25*†
**Height** (cm)	136.09±6.14	153.87±4.96*	167.65±5.47*†
**Body Mass** (kg)	32.66±6.18	45.54±6.57*	62.00±8.27*†
**APHV** (years)	12.15±0.77	12.63±0.66	13.38±0.92*†
**Maturity Offset**	-2.81±1.0	-0.21±0.57	2.01±0.57*
**GMS**	83.62±6.08	74.25±12.91	83.62±6.09*†
**Object Control**	41.21±3.99	34.63±5.95	41.21±4.00*
**Locomotor**	42.40±3.12	39.63±7.29	42.40±3.12†

*Significantly different from pre-PHV

†Significantly different from peri-PHV

µSignificantly different from post-PHV

Abbreviations: age of peak height velocity (APHV), peak height velocity (PHV)
